# Penetrant PKCβ mutation in ATLL displays a mixed gain-of-function

**DOI:** 10.1042/BCJ20253384

**Published:** 2025-11-04

**Authors:** Sophie JL. Brown, David C. Briggs, Patrick Costello, Hiroko Yaguchi, Charles RM. Bangham, Peter J. Parker, Neil Q. McDonald

**Affiliations:** 1 Protein Phosphorylation Laboratory; 2 Signalling and Structural Biology Laboratory; 3 Signalling and Transcription Laboratory, Francis Crick Institute, London, U.K.; 4 Department of Infectious Diseases, Faculty of Medicine, Imperial College London, London, U.K.; 5 School of Cancer and Pharmaceutical Sciences, Guy’s Campus, King’s College London, London, U.K.; 6 Institute of Structural and Molecular Biology, School of Natural Sciences, Birkbeck College, London, U.K.

**Keywords:** ATLL, cancer associated mutation, HTLV-1, PKCβ

## Abstract

Mutations in the T-cell receptor signalling pathway have been identified in patients with adult T-cell leukaemia/lymphoma (ATLL) and one of the most frequently observed targets of these mutations is protein kinase C beta (PKCβ). Here, we have characterised the most frequent mutation in PKCβ (D427N), addressing the issue of gain/loss of function, neomorphic change and assessing the impact of mutation *in vivo*, in cells, biochemically and structurally. It is concluded that this mutation is a gain-of-function, activating mutation that confers an altered substrate specificity on this protein kinase. In a constitutive knock-in mouse model, this activated allele induces splenomegaly associated with extramedullary haematopoiesis. Pharmacologically, the D427N mutant protein displays poor sensitivity to established PKCβ inhibitors, necessitating the development of bespoke therapeutics for any ATLL intervention through this target. Such efforts could be guided by the availability of the D427N mutant-ruboxistaurin structure presented here.

## Introduction

Human T-cell leukaemia virus type 1 (HTLV-1), which chiefly infects CD4^+^ T-cells, is associated with adult T-cell leukaemia/lymphoma (ATLL), a rare and aggressive T-cell malignancy [1,2]. With a long latency period (30–60 years), ATLL develops in 5% of HTLV-1 carriers [2], and median survival rates of the disease remain poor (less than one year for the acute and lymphoma subtypes) [3–5]. There have been limited epidemiological studies in regions where HTLV-1 is endemic; nevertheless, it is estimated that at least 10 million people are infected worldwide [6].

Integration of the HTLV-1 provirus into the T-cell genome gives rise to typically >10^4^ unique HTLV-1-infected T-cell clones in HTLV-1 carriers [7]. Two viral proteins, HBZ and Tax, play important roles in ATLL oncogenesis by various mechanisms, including activation of NF-κB [8]. As a target of the host cytotoxic T-cell response, Tax is expressed in rare ‘bursts’, whereas HBZ, which is not strongly immunogenic, is expressed in infected T-cells 50% of the time [9]. Co-operation between Tax and HBZ enables infected T-cell clones to persist in the host over decades, and it has been proposed that accumulation of mutations in long-lived infected T-cell clones, possibly due to mitotic errors, is the main mechanism of ATLL oncogenesis [2].

A genomic sequencing study of 426 ATLL cases [10] first identified the most frequently occurring somatic mutations, showing that more than 90% of ATLL patients have mutations in the components of the TCR-NF-κB signalling pathway. Known or predicted gain-of-function mutations are found in positive regulators of TCR-NF-κB signalling, and loss-of-function mutations are found in negative regulators. For example, *PLCG1* was the most frequently mutated gene (36%) identified and has an activating hotspot [11]; gain-of-function mutations were also found in *CARD11* (24%). The second most frequently mutated gene in this cohort of patients was *PRKCB* (33%), encoding protein kinase C beta (PKCβ); interestingly, this was the most frequently mutated gene (43.8%) in a recent study of an Okinawa cohort [12]. Whilst PKCβ functions downstream of the B-cell receptor to activate NF-κB in B-cells [13,14], it is not essential in T-cells [15]. By contrast, PKCθ is the isoform well characterised as functioning in T-cell receptor signalling [16]; it is unclear whether PKCβ can act in its place or is, for example, associated with feedback control.

Like all PKCs, PKCβ has an N-terminal regulatory domain and a C-terminal catalytic domain and, in the absence of activators, adopts an autoinhibited conformation in which an N-terminal pseudosubstrate sequence is bound to the catalytic domain [17]. PKCβ is also alternatively spliced to give rise to two isoforms, PKCβI and PKCβII, which differ by one exon, encoding the kinase domain C-terminal tail [18,19] . As typical of all conventional PKC (cPKC) isoforms, increases in cytoplasmic calcium and plasma membrane diacylglycerol cause PKCβ to relocate from the cytoplasm to the plasma membrane; the pseudosubstrate sequence is released under these circumstances, resulting in an open PKC conformer [20]. The observation of activating mutations in *PLCG1* responsible for generation of these PKCβ triggers is compatible with the view that the ATLL mutations lie on a pro-proliferative T-cell pathway [10].

**Figure 1:**
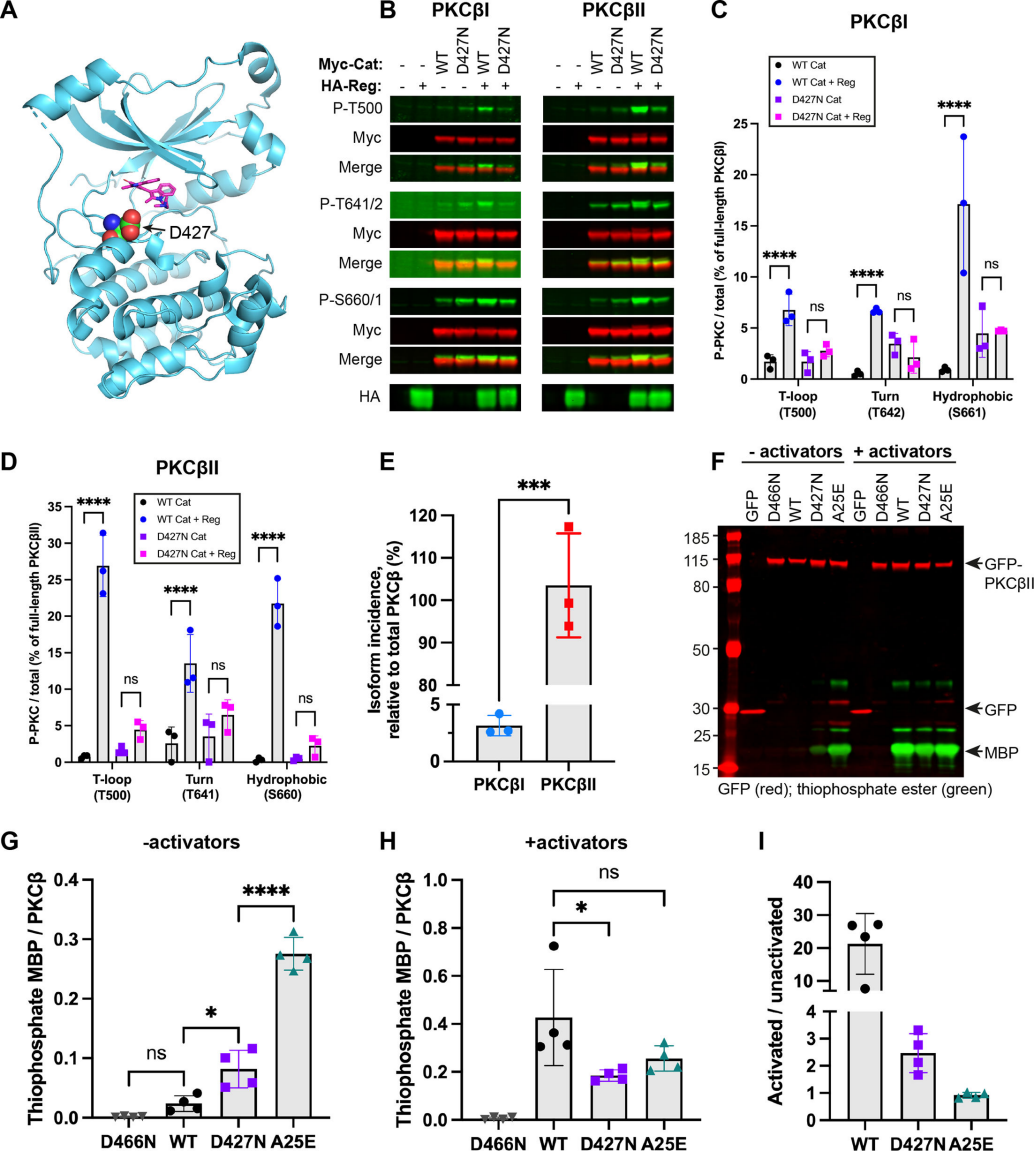
D427N PKCβ expressed in mammalian cells is a partially open and active conformer. **A** – Crystal structure of WT PKCβII bound to 2-methyl-BIM-1 (PDB 2I0E) (Grodsky et al.) showing the position of D427. **B** – Western blots of lysates from HEK293 cells transfected with the indicated Myc-PKCβ catalytic domain constructs ± HA-PKCβ regulatory domain, probed with antibodies against Myc, the three phosphorylated PKC priming sites and HA. Blots are representative of three biological replicates. **C & D** – Quantification of priming of PKCβI and PKCβII from the blots in (**B**). For each priming site and construct, the ratio of phosphorylated PKC to Myc was calculated and converted to a % priming of full-length PKCβI or PKCβII. Error bars indicate the mean ± SD (*n*=3, ****=*P*<0.0001, two-way ANOVA followed by Tukey’s post-hoc test). **E** – % isoform incidence of PKCβI and PKCβII mRNA in Jurkat cells by qPCR, calculated from the fold change in expression relative to total PKCβ (2^−ΔCT^ × 100). Error bars indicate the mean ± SD (*n*=3, ***= *P*<0.001 by unpaired t-test). **F** – Western blot of GFP-PKCβII immunoprecipitation kinase assays towards MBP ± activators, probed with antibodies against GFP and thiophosphate ester. The blot is representative of four biological replicates. **G & H** – Quantification of thiophosphorylated MBP/total PKCβ from (**F**). Error bars indicate the mean ± SD (*n*=4, *=*P*<0.05, ****=*P*<0.0001 by one-way ANOVA with Tukey’s correction for multiple comparisons). **I** – Activated/unactivated ratios from (**G**) and (**H**). MBP, myelin basic protein.

Of the PKCβ mutations identified in ATLL, the majority are in the catalytic domain, and there is a prominent hotspot at D427. D427N is the most penetrant mutation, comprising ~70% of ATLL PKCβ mutations [10]. D427 is in the lobe linker of the kinase domain, close to both the ATP-binding and substrate-binding sites (see below Figure 1A) [21]. D427 is the first residue of α-helix D in PKCβ, referred to as the ‘αD1’ position [22]. Conserved as aspartate or glutamate across AGC kinases, the αD1 residue has a likely role in substrate binding: for example, in a crystal structure of PKCι, the αD1 aspartate contacts arginine at P-3 of a PKC consensus substrate, which also lies close to the ribose hydroxyl of the bound nucleotide [23]. It has been suggested that the D427N mutation could be a neomorphic mutation, ‘rewiring’ the substrate specificity away from arginine at P-3 [22]. Assuming that the pseudosubstrate follows a similar trajectory to a substrate, D427N could also be a gain-of-function mutation, activating the kinase by disrupting binding of the pseudosubstrate; indeed, there is evidence that D427N PKCβ increases NF-κB activation in Jurkat cells [10]. It has also been suggested that by inducing an open conformation, the D427N mutation could promote the degradation of PKCβ and result in loss of function [24].

Given the mutational penetrance, the association with more aggressive subtypes of ATLL [12,25] and the druggability of this protein kinase [26,27], D427N PKCβ offers itself as a potentially important therapeutic target. However, the effects of the D427N mutation on the properties of PKCβ (gain/loss of function) and the role of D427N PKCβ in ATLL oncogenesis are not clear, and a better understanding is required to inform any therapeutic intervention. Here, we assessed the structure and properties of D427N PKCβ *in vitro*, in cell-based assays and in mice. Our findings support inhibition of D427N PKCβ activity as a potential treatment for ATLL.

## Results

### Conformation and activity of D427N PKCβ expressed in mammalian cells

The isolated PKCβII catalytic domain is not phosphorylated at its three priming sites, but priming can be partially restored by co-expression of the regulatory domain [28]. This property was exploited to compare the regulatory-catalytic domain interaction of WT and D427N PCKβI/βII proteins, determining the priming stoichiometry of each catalytic domain with or without regulatory domain co-expression in HEK293 cells (Figure 1B). Priming of WT PKCβI/βII catalytic domains was significantly increased at all three sites in the presence of the regulatory domain, as expected (Figure 1C & D). This was not the case for D427N PKCβI/βII catalytic domains, indicating reduced regulatory-catalytic domain interaction and by inference, a more open/active conformation for the full-length protein (Figure 1C & D). In agreement with this, experiments showed significantly increased basal phosphorylation of PKC substrates in HEK293 cells transfected with D427N PKCβI compared with WT PKCβ; by comparison, there was no significant difference when cells had been treated with the PKC activator phorbol-12-myristate 13-acetate (PMA) (Supplementary Figure S1A and B).

To determine whether to focus on PKCβI or PKCβII, we compared the relative expression of PKCβI vs PKCβII mRNA in Jurkat T-cells by qPCR. The majority (>90%) of PKCβ mRNA in Jurkat T-cells encoded PKCβII (Figure 1E) and this was not due to any difference in primer amplification efficiency (Supplementary Figure S1C). mRNA sequencing of peripheral blood mononuclear cells (PBMCs) from two ATLL patients also showed PKCβII was the major isoform, albeit to a lesser extent (Table 1). Given it is the dominant form, we focused our studies on PKCβII.

**Table 1:** PKCβI and PKCβII transcripts per million in RNA sequencing data from peripheral mononuclear blood cell samples of two ATLL patients

Patient	Transcripts per million (TPM)		PKCβI (%)	PKCβII (%)
	PKCβI (ENST00000321728)	PKCβII (ENST00000643927)		
A	29.1 ± 2.9	42.0 ± 6.5	41.1 ± 2.0	58.9 ± 2.0
B	11.4 ± 1.2	14.9 ± 2.8	43.7 ± 6.8	56.3 ± 6.8
Mean ± SD	23.2 ± 9.2	33.0 ± 14.5	42.0 ± 4.0	58.0 ± 4.0

Based on the pattern of regulatory-catalytic domain interactions, the autoinhibition of D427N PKCβ appears to be reduced; hence, we compared the activity of WT and D427N PKCβII in the presence and absence of activators, alongside D466N (kinase-dead) and A25E (a constitutively active pseudosubstrate mutant) PKCβII controls. GFP-tagged proteins were immunoprecipitated from HEK293 cells and activities assayed with myelin basic protein (MBP) and ATPγS as substrates (Figure 1F). In the absence of activators, D427N PKCβII had significantly higher activity than WT PKCβII but lower activity than A25E PKCβII, indicating that D427N PKCβII is not a fully open, constitutively active conformer but is partially open/active under basal conditions (Figure 1G). In the presence of activators, D427N PKCβII had significantly lower activity than WT PKCβII towards MBP (Figure 1H). Independent of any substrate specificity variation, the activated/basal ratio showed that WT PKCβII activity was highly dependent on activators (20-fold), D427N PKCβII retained a much reduced dependency on activators (2.5-fold), and A25E PKCβII was not dependent on activators for activity (Figure 1I), consistent with an open/activated conformation for the D427N mutant.

### Activity of recombinant D427N PKCβ

To compare the intrinsic activities of WT and mutant proteins directly, WT PKCβII and D427N PKCβII were expressed in and purified from SF21 cells (Supplementary Figure S2A-D). Full-length D427N PKCβII was more prone to aggregation than WT, evidenced by the greater proportion of material consistently observed in the void peak (Supplementary Figure S2A and B). We compared the priming stoichiometry of purified proteins by western blotting with antibodies for each phosphorylated PKC priming site (Supplementary Figure S2E and F) and also by tandem mass spectrometry (Supplementary Table S2). This confirmed that these purified proteins were consistently primed at each site, although a small reduction in priming of the WT PKCβII T-loop was observed by mass spectrometry (Supplementary Table S2).

**Figure 2:**
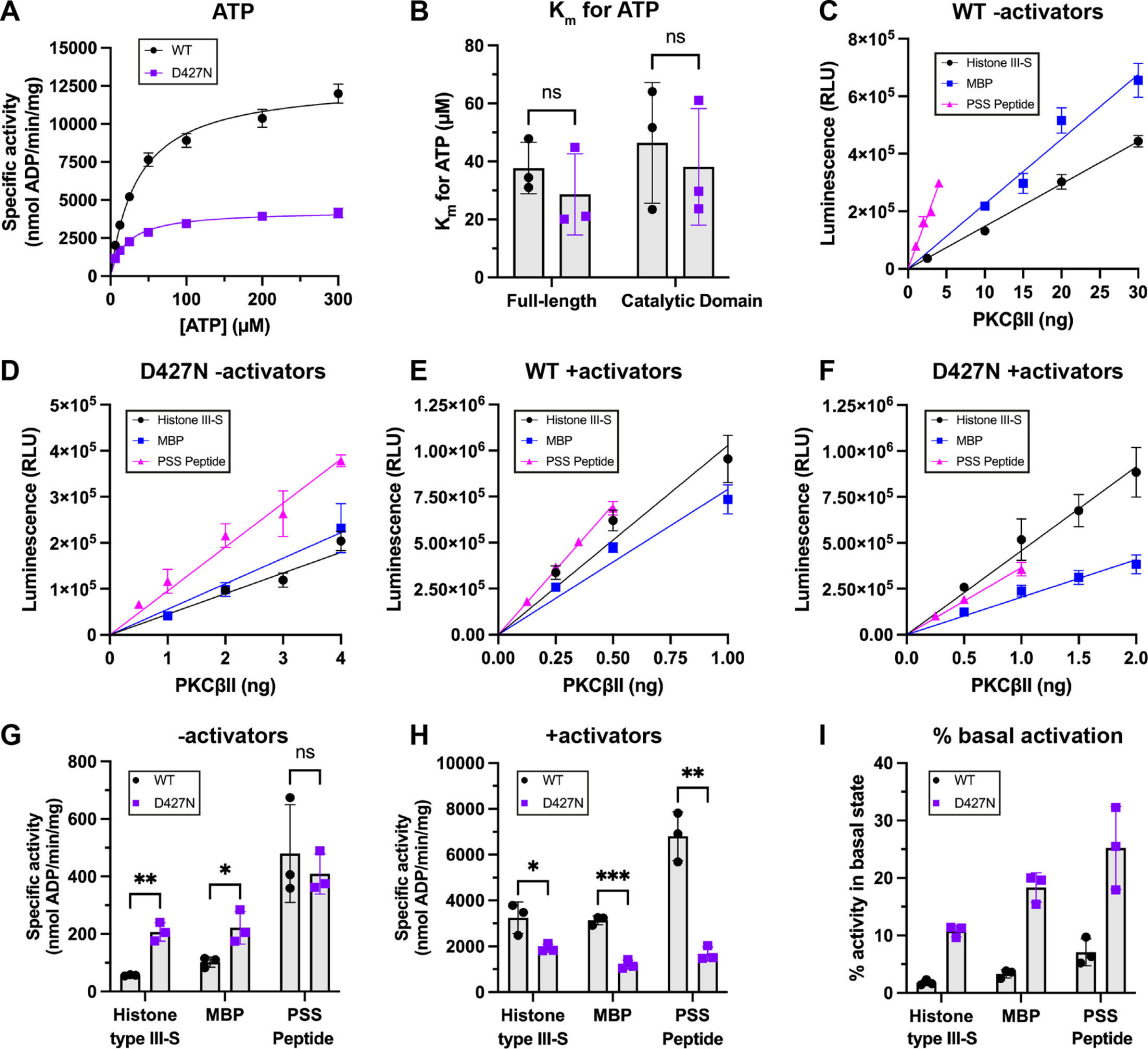
*In vitro* activity of WT and D427N PKCβII towards cPKC substrates in the presence and absence of activators. **A** – Representative titration curves when full-length WT or D427N PKCβII were assayed with excess histone type III-S and a titration of ATP. PMA activator was present. Error bars indicate the mean ± SD from three technical replicates. **B** – K_m_ for ATP of WT and D427N PKCβII (full-length and catalytic domains) obtained from Michaelis–Menten fits of ATP titration curves. Error bars indicate the mean ± SD from three independent assays (ns by unpaired t-test). **C–F** – Representative plots of luminescence vs WT or D427N PKCβII quantity when assayed with 50 μM ATP and either PSS peptide (200 μM), histone type III-S (50 μM) or MBP (50 μM), in the presence and absence of activators. Error bars indicate the mean ± SD from three technical replicates. **G & H** – Specific activity values of WT and D427N PKCβII towards each substrate ± activators. Specific activity in nmol/min/mg was calculated from the gradient of the straight lines in (**C-F**) using an ADP standard curve. Error bars indicate the mean ± SD from three independent assays (*=*P*<0.05, **=*P*<0.01, ***=*P*<0.001 versus WT by t-test with Holm–Šídák correction for multiple comparisons). **I** – % of full activity without activators, calculated from (**G**) and (**H**).

To compare activities and specificities *in vitro*, we first determined the K_m_ for ATP of WT and D427N PKCβII. There was no significant difference between the K_m_ for ATP for these proteins, and this was true of both full-length proteins (Figure 2A) and their isolated catalytic domains (Figure 2B). To assess the activation state of the full-length recombinant proteins and their specific activity against canonical cPKC substrates [MBP, histone type III-S and a peptide based upon the PKCβ pseudosubstrate sequence containing a single phosphorylation site (PSS peptide)], we titrated full-length WT and D427N PKCβII proteins at fixed ATP (50 μM) and excess substrate concentrations (Figure 2C–F). In the absence of activators, D427N PKCβII had significantly higher specific activity compared with WT for both MBP and histone type III-S; there was no significant difference for the PSS peptide (Figure 2G). In the presence of activators, D427N PKCβII had significantly lower specific activity for all three substrates (Figure 2H). In agreement with the immunoprecipitation-kinase assays (Figure 1), the basal activity of D427N PKCβII was 10–25% of its full activity depending on the substrate, indicating D427N PKCβII is partially open and active (Figure 2I). The similar specific activity of WT and D427N PKCβII towards PSS peptide in the absence of activators (Figure 2G) likely reflects a combination of the lesser dependency of WT PKCβII for activators in the context of a peptide substrate (Figure 2I), and the lower intrinsic activity of D427N PKCβII towards this peptide (Figure 2H).

### Substrate specificity of D427N PKCβ

**Figure 3:**
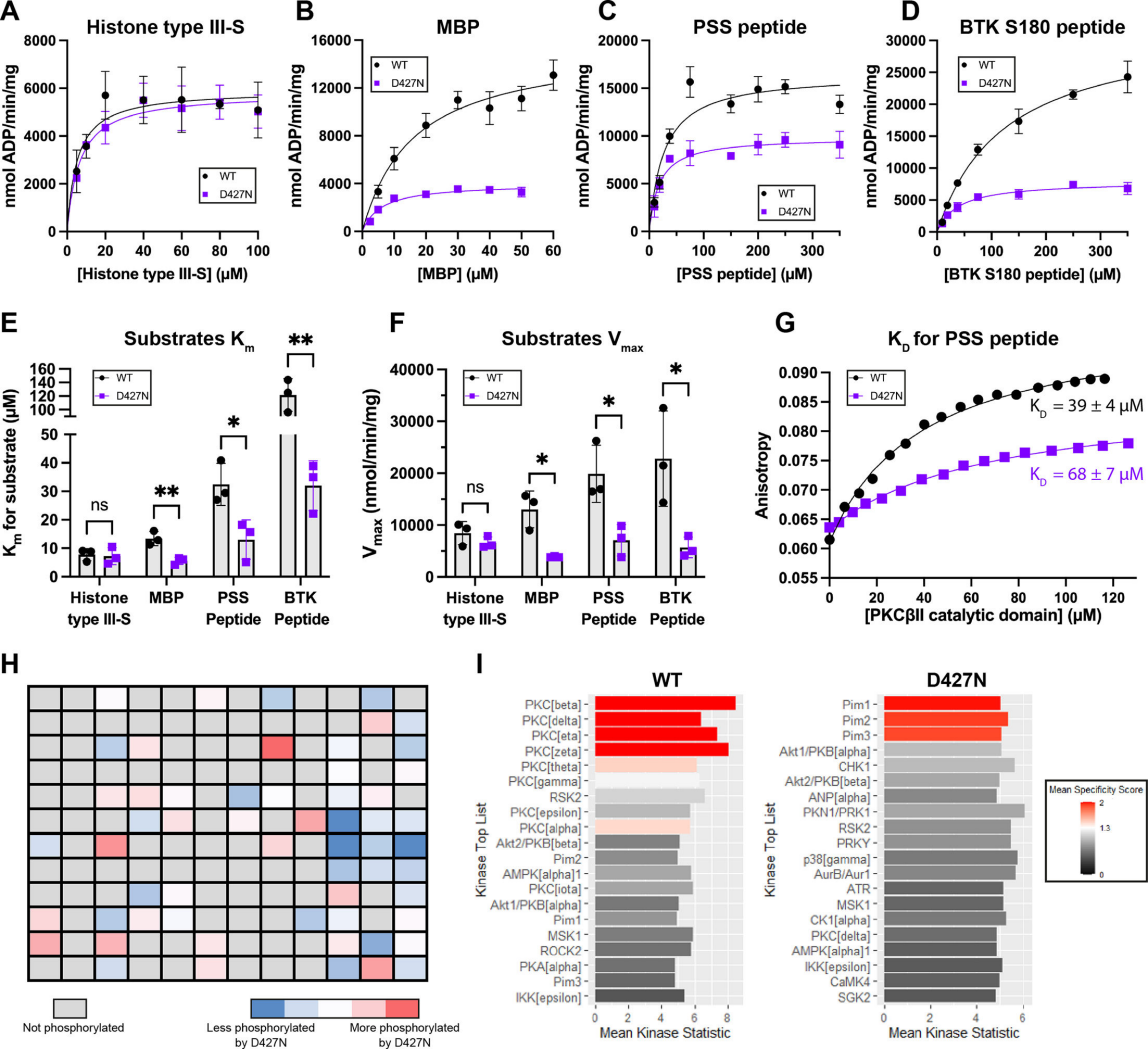
D427N PKCβII has an altered substrate repertoire *in vitro* **.** **A–D** – Representative titration curves from assays of WT or D427N PKCβII catalytic domains with excess ATP and titrations of either histone type III-S, MBP, PSS peptide or BTK S180 peptide. Specific activity in nmol/min/mg was calculated using an ADP standard curve. Error bars indicate the mean ± SD from three technical replicates. **E and F** – K_m_ and V_max_ values of WT and D427N PKCβII catalytic domains for each substrate, obtained from Michaelis–Menten fits of titration curves in (**A-D**). Error bars indicate the mean ± SD from three independent assays (*=*P*<0.05, **=*P*<0.01 versus WT by t-test with Holm–Šídák correction for multiple comparisons). **G** – Representative binding curves of anisotropy vs protein concentration when WT or D427N PKCβII catalytic domains were titrated into fluorescein–PSS peptide; K_D_ values are shown ± the error of the fit. WT means K_D_=35.3 ± 4 μM (*n*=3) and D427N means K_D_=72 ± 11 μM (*n*=2). **H** – Differential heat map of WT and D427N PKCβII activity towards a PamChip microarray, calculated from log2 fold change values relative to a no ATP control array. **I** – Upstream kinase analysis of the PamChip microarrays. Plots show a list of the top kinases predicted to have phosphorylated the array alongside the ‘mean kinase statistic’ and colour-coded according to the ‘specificity score’. The specificity score represents the specificity of the change in kinase activity relative to the no ATP control (higher specificity score=lower chance the result could have occurred with a random set of peptides). The mean kinase statistic represents the change in kinase activity on the array (>0=activation; this metric is more relevant to analysis of cell lysates).

Given the possibility that D427N is a network-rewiring mutation [22], the lower specific activity of D427N PKCβII towards cPKC substrates in the presence of activators suggested that D427N PKCβ may have altered substrate specificity. To compare the K_m_ and V_max_ of WT and D427N PKCβII towards cPKC substrates, we assayed the catalytic domains with a titration of MBP, histone type III-S, PSS peptide and also a peptide containing the PKCβ phosphorylation site in Bruton’s tyrosine kinase (BTK S180) [29]) (Figure 3A–D). D427N PKCβII had a significantly lower K_m_ and lower V_max_ than WT for all substrates tested apart from histone type III-S, for which the K_m_ and V_max_ were not significantly different (Figure 3E & F). These results suggest that, whilst D427N PKCβII retains the same catalytic potential as WT, evidenced by the similar activity towards histone type III-S, it does not phosphorylate other cPKC substrates as efficiently as WT. The consistently lower K_m_ values for D427N PKCβII were unexpected, suggesting the mutant may bind these substrates with higher affinity. As K_m_ is not a direct measure of binding affinity, we used fluorescence polarisation to obtain binding curves for WT and D427N PKCβII catalytic domains towards the PSS peptide (Figure 3G). The resulting K_D_ values suggest that, unlike the K_m_ values, D427N PKCβII binds PSS peptide with two-fold weaker affinity than WT (consistent with the reduced regulatory domain interaction). It is surmised that the lower apparent K_m_ reflects the fact that for PKCβII, K^−1^ is not >>k_cat_ and hence a reduced k_cat_ impacts the apparent K_m_.

To investigate in an agnostic manner whether D427N PKCβII has an altered primary sequence preference towards substrates compared with WT, we assayed WT and D427N PKCβII catalytic domains on a PamChip® microarray containing 144 phosphorylation sites found in a diverse set of serine/threonine kinase substrates (Supplementary Figure S3). Log fold change values relative to no-ATP control arrays were used to generate a differential heat map for WT and D427N PKCβII, illustrating distinctive patterns of phosphorylation of this set of substrates (Figure 3H). These data were probed by an upstream kinase analysis, in which databases of known kinase substrate relationships were used to predict which kinases would correlate with the patterns of phosphorylation observed for WT and D427N PKCβ. This indicated that, whilst the pattern of peptides phosphorylated by purified WT PKCβII matched well with the PKC family (WT PKCβ was the top pattern hit), the pattern of peptides phosphorylated by the D427N PKCβII catalytic domain matched most closely with the specificity of PIM kinases (Figure 3I). These results suggest that the D427N mutation does indeed alter the potential substrate repertoire of PKCβ.

### Activity of D427N PKCβ *in vivo*

Taken together, the partial activation of D427N PKCβ under basal conditions and evidence of altered substrate repertoire suggested that D427N PKCβ has mixed gain-of-function properties. To determine the effect of these altered properties on T-cells *in vivo*, we generated a mouse model heterozygous for the D427N mutation in all tissues, by knock-in of the mutation into the endogenous *Prkcb* gene locus. Immunophenotyping of the spleen, thymus, bone marrow and inguinal lymph nodes of D427N PKCβ heterozygotes (hets) and WT littermates at 12 weeks and 1 year of age showed no significant differences between the proportions of common T-cell populations. Therefore, as ATLL is a disease with a long latency period, we continued to monitor a cohort of D427N PKCβ hets and WT littermates for the development of a phenotype up to 18 months of age. Within this cohort, 14 animals were culled early consequent to overgrooming and related behaviours (five hets and nine WT) and two hets with spleen phenotypes were culled (see further below).

**Figure 4:**
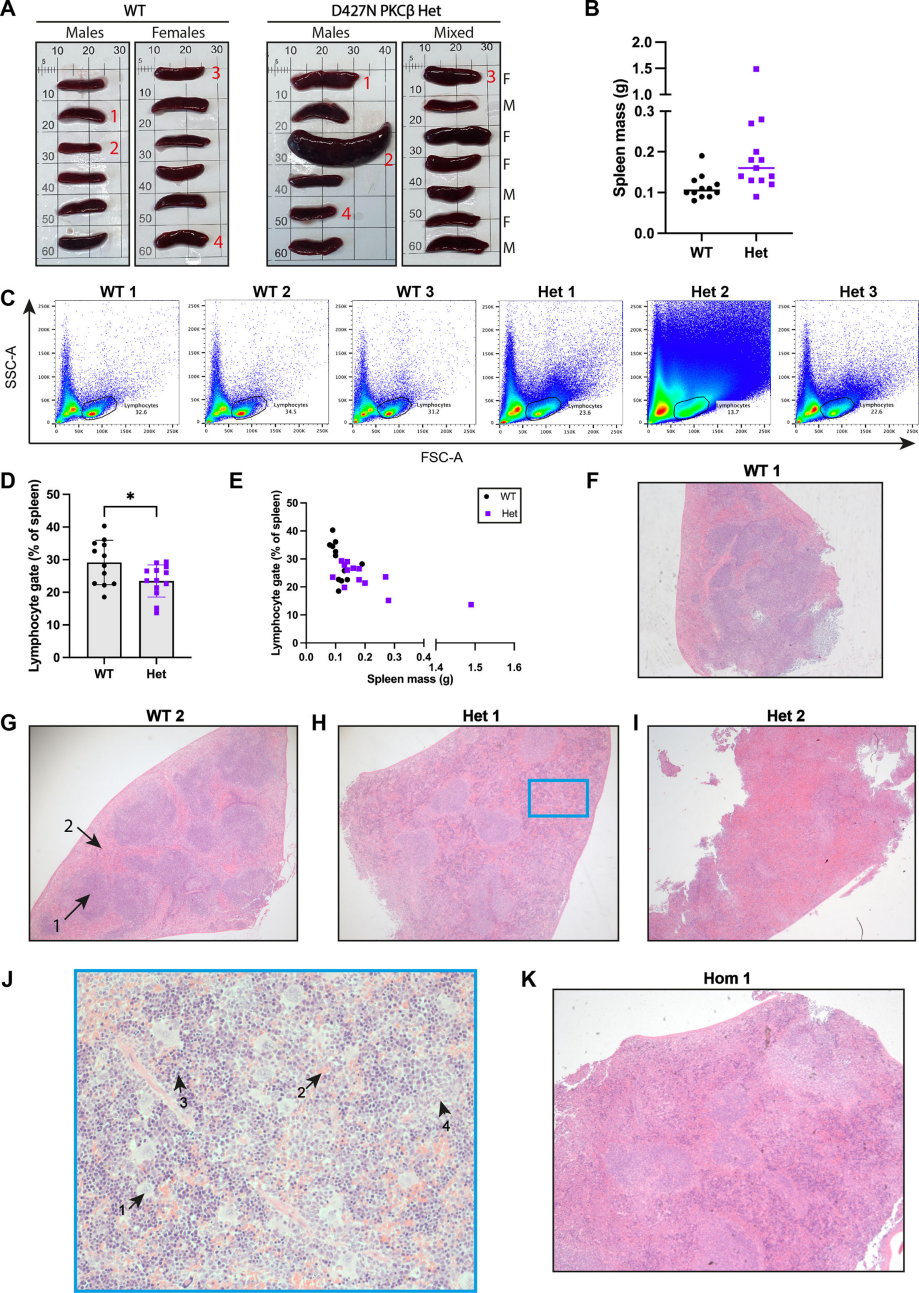
Development of a phenotype in the spleen of a D427N PKCβ mouse model. **A** – Images of spleens harvested from WT and D427N PKCβ hets at 18 months old. **B** – Masses of the spleens in (**A**), showing the median mass. **C** – Examples of SSC-A vs FSC-A plots obtained from the numbered spleens in (**A**), showing the lymphocyte gate. **D** – % of lymphocytes in the spleen as gated by SSC-A vs FSC-A. Error bars indicate the mean ± SD from the 12 WT mice and 13 D427N PKCβ hets in (**A**) (*=*P*<0.05 by unpaired t-test). **E** – % of lymphocytes from (**D**) plotted against spleen masses from (**B**). **F–I** – Images of haematoxylin and eosin-stained spleen sections from the numbered spleens in (**A**). **J** – Higher magnification image of the indicated region in (**H**). Arrow 1=megakaryocyte; arrow 2=erythrocytes; arrow 3=erythroid precursor; arrow 4=granulocyte precursor. **K** – Image of a haematoxylin and eosin-stained spleen section from a D427N PKCβ hom culled sick at eight months.

Notwithstanding the above exceptions, we harvested the spleens of 13 D427N PKCβ hets and 12 WT littermates which survived to 18 months with no health concerns. The spleen masses showed that a subset of D427N PKCβ hets had splenomegaly compared with WT; in most cases, this was a 2–3-fold enlargement, and it occurred in both male and female mice (Figure 4A & B). We carried out immunophenotyping of a cell suspension from each spleen by flow cytometry. SSC-A vs FSC-A plots from D427N PKCβ hets with splenomegaly showed much more variation in cell size and granularity than WT (Figure 4C). Indeed, the proportion of cells within the lymphocyte population, as gated by SSC-A and FSC-A, was significantly reduced in D427N PKCβ hets compared with WT (Figure 4D) and the two most-enlarged spleens had the greatest reduction (Figure 4E). FACS analysis showed no significant difference in the proportions of CD4 or CD8 single-positive T-cells in the spleens of WT vs D427N PKCβ hets, but there was a significant reduction in the proportion of B220+ B cells in D427N PKCβ hets (Supplementary Figure S4). To determine which cell populations were expanded in these spleens, we analysed haematoxylin and eosin-stained spleen sections from four D427N PKCβ hets and four WT mice (Figure 4F–I). Three D427N PKCβ hets with splenomegaly scored more highly than WT for extramedullary haematopoiesis (EMH) (Table 2), diagnosed by an increased volume of red pulp and increased quantity of haematopoietic stem cells in the red pulp. Haematopoietic stem cells from the megakaryocytic, erythroid and myeloid lineages were visible in these D427N PKCβ hets (Figure 4J). Increased EMH would explain the cell populations with increased FSC-A and SSC-A and reduced proportion of lymphocytes overall in these spleens.

**Table 2:** Semi-quantitative grading of haematoxylin and eosin-stained mouse spleen sections. 0=not present; 1=minimal; 2=mild; 3=moderate; 4=marked; *P*=present and not gradable. The spleens were from four WT and four D427N PKCβ hets at 18 months old (Hets 1–3 had splenomegaly and a phenotype by flow cytometry, whereas Het 4 did not; see spleen images in Figure 4). Hom 1 was culled sick at eight months and had splenomegaly.

Feature	WT 1	WT 2	WT 3	WT 4	Het 1	Het 2	Het 3	Het 4	Hom 1
EMH	1	1	2	2	3	3	3	2	4
Haemosiderin	1	1	3	3	1	1	3	2	2
Increase in white pulp	0	0	0	0	0	0	0	0	0
Secondary follicles	P	0	P	P	0	P	0	P	P
Congestion	0	0	0	0	0	P	0	0	0

Out of a more limited cohort of mice homozygous for the D427N PKCβ mutation at the endogenous *Prkcb* locus, 5/7 mice were culled sick between 22 and 32 weeks. We analysed the spleens of two of these D427N PKCβ homs (one culled due to seizures and one due to a fighting wound), and both showed a similar phenotype by flow cytometry as observed for the 18-month D427N PKCβ hets. Indeed, haematoxylin and eosin staining of a fixed spleen section from one of these D427N PKCβ homs showed it had the highest possible score for EMH (Figure 4K and Table 2). Evidence of this phenotype at this earlier age in D427N PKCβ homozygotes suggests that it does arise from D427N PKCβ expression, either directly in the myeloid lineage or as a by-product of other stresses in D427N PKCβ mice.

### Pharmacology of D427N PKCβ

**Figure 5:**
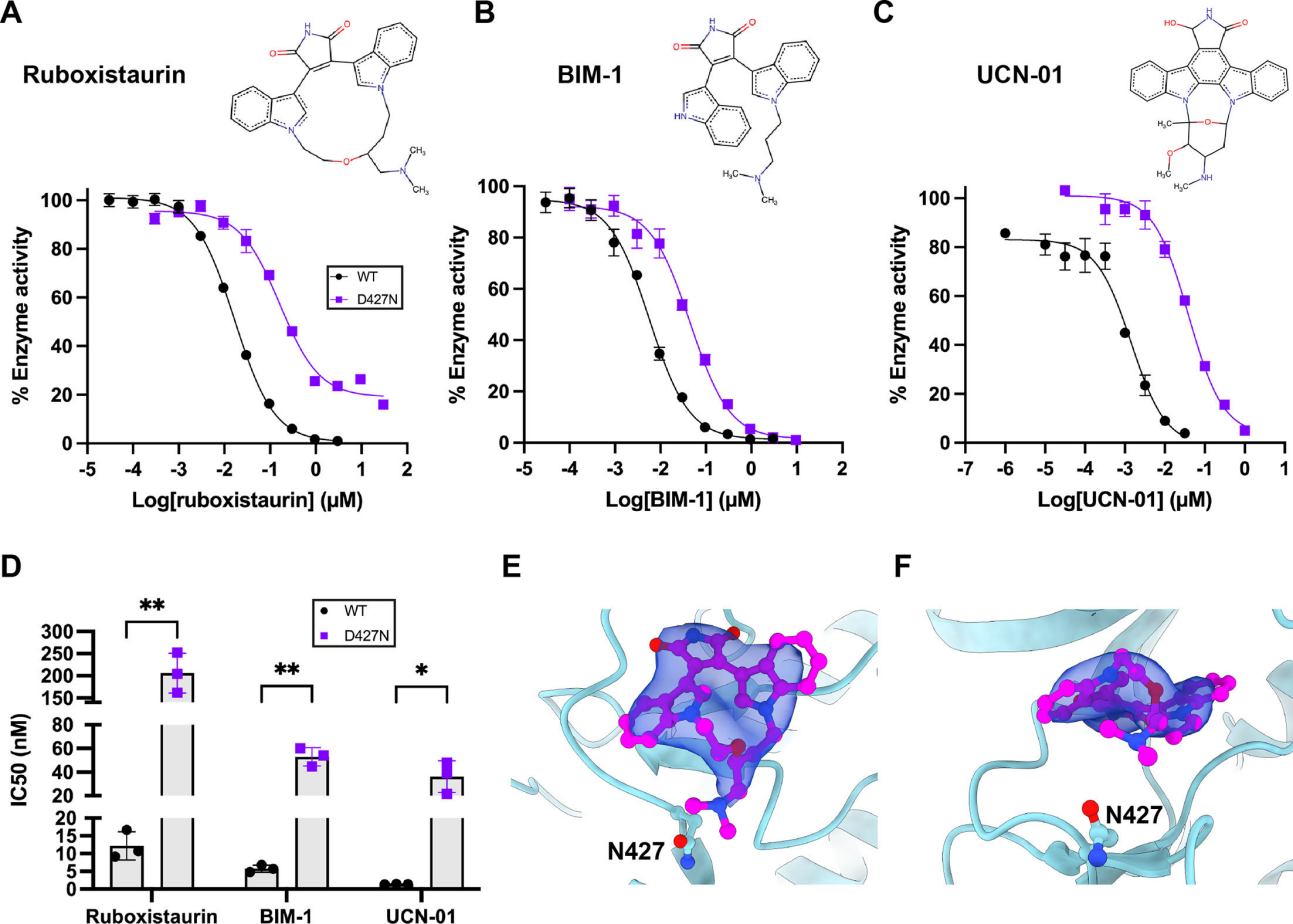
Pharmacology of D427N PKCβII. **A–C** – Representative dose-response curves when WT and D427N PKCβII catalytic domains were assayed with the indicated inhibitors, 50 μM ATP and 200 μM PSS peptide. ADP produced in the assays was measured by luminescence and converted to specific activity using an ADP standard curve. Error bars indicate the mean ± SD from three technical replicates. **D** – IC50 values obtained from fitting curves in (**A-C**). Error bars indicate the mean ± SD from three independent dose-response assays (*=*P*<0.05, **=*P*<0.01 versus WT by t-test with Holm–Šídák correction for multiple comparisons). **E,F** – Orthogonal views of Ruboxistaurin (pink) in the PKCβII nucleotide-binding pocket (cyan). A σA-weighted |2Fo-Fc| map is shown contoured at 1.5 σ. The location of the N427 side chain is indicated. Figure was generated using UCSF ChimeraX.

The mixed gain-of-function biochemical properties of D427N PKCβ and the dysplastic phenotype *in vivo* suggest that PKCβ may be a target for inhibition in ATLL. Given the proximity of the mutation to the nucleotide pocket of the kinase, we compared the potency of three ATP-competitive inhibitors in WT and D427N PKCβII to assess if existing PKC inhibitors could be used to target the mutant. We assayed WT and D427N PKCβII catalytic domains at 50 μM ATP with one indolocarbazole inhibitor (UCN-01) and two bisindolylmaleimide inhibitors (BIM-1 and the PKCβ-specific inhibitor ruboxistaurin) (Figure 5A–C). For all three inhibitors, the IC50 for the D427N PKCβII catalytic domain mutant was substantially higher than WT, suggesting that the mutant has weaker affinity for these inhibitors (Figure 5D). These differences were 17-fold for ruboxistaurin, 9-fold for BIM-1 and 25-fold for UCN-01. These results show that it is unlikely existing inhibitors could be used to target D427N PKCβ catalytic activity successfully.

The β-selective ruboxistaurin is one of the very few isoform-specific PKC inhibitors [27], and we sought to understand the molecular interaction between this drug and the PKCβII catalytic domain to gain insight into the idiosyncrasies of the D427N PKCβII mutant. We determined the crystal structure of ruboxistaurin bound to the D427N PKCβII catalytic domain at 3.4 Å (Supplementary Table S2). The determined structure has the classical eukaryotic protein kinase fold and C-terminal tail characteristic of an AGC kinase (Figure 5E). There was electron density missing from A626 to P637 in the C-terminal tail, indicating this region is disordered, but N628 of the NFD motif was visible, showing the NFD motif in an ‘outward’ conformation, facing away from the nucleotide cleft; in ATP-bound kinase structures the phenylalanine side chain of this conserved motif contacts the adenine ring of ATP [30]. The activation loop of D427N PKCβII is ordered, and electron density showed it was phosphorylated at T500; there was also clear electron density showing phosphorylation of the other two PKC priming sites in the C-terminal tail. At the base of the activation loop, the DFG motif is in an ‘inward’ conformation, associated with an active kinase, and the catalytic aspartate (D466) is oriented towards the nucleotide pocket. Although 3.4 Å is medium-low resolution, the electron density map was of good quality, as evident around the priming sites.

Electron density within the nucleotide pocket was consistent with a bound ruboxistaurin compound. The indole rings are observed to adopt a non-planar conformation, and the maleimide group of the inhibitor contacts the main chain of two residues in the hinge region (E421 and V423). The glycine-rich loop is collapsed onto the inhibitor, and the phenylalanine side chain packs against the indole rings. In the hinge region, the side chain of N427 is orientated towards the nucleotide pocket, in closest proximity to the dimethylamine group of the inhibitor. Electron density for the dimethylamine group was not well resolved, suggesting it is mobile.

The binding of ruboxistaurin to the D427N PKCβII catalytic domain was compared with a structure of WT PKCβII bound to 2-methyl-BIM1 [21]. The overall backbone of the two structures is very similar; both have ordered activation loops and are phosphorylated at all three priming sites. WT PKCβII has an additional α-helix containing the NFD motif, whereas in D427N PKCβII this region is disordered. In the nucleotide pocket, one of the indole rings of each inhibitor is in an identical position, packed against the F353 side chain of the collapsed glycine-rich loop, but the other indole ring in 2-methyl-BIM-1 is ‘flipped’ compared with ruboxistaurin, indicating a distinctive binding pose. The maleimide groups of both inhibitors are in a similar position and make polar contacts with the main chain of E421 and V423 in the hinge region. However, the maleimide group of 2-methyl-BIM-1 is slightly shifted and makes an additional polar contact with T404; this is not the case in the D427N PKCβII model, as the side chain of T404 is in a different orientation. The other main difference in the binding of the two inhibitors is that in the WT PKCβII structure, D470 contacts the dimethylamine group of the inhibitor; in the D427N PKCβII model, the side chain of D470 is in a different orientation, and this contact is not made. Although the two inhibitors are different, the additional polar contacts between WT PKCβII and 2-methyl-BIM-1 likely contribute to the higher affinity of WT PKCβII for this class of ATP-competitive inhibitors compared with the mutant. D427 and N427 are in an identical position in the two structures with the side chains orientated towards the nucleotide pocket, suggesting that the D427N mutation does not induce large conformational changes but influences the local environment of the nucleotide and substrate binding pockets.

## Discussion

The evidence presented here demonstrates that the D427N PKCβ mutation commonly found in ATLL patients is an activating mutation that also modifies the mutant kinase’s substrate repertoire. Whilst other mutations in ATLL commonly involve the TCR–NFκB pathway [10], it remains to be seen exactly how the altered D427N PKCβ properties confer its driver action. However, irrespective of the underlying molecular action, it is anticipated that intervention in the poor prognosis, *PRKCB*-mutant form of ATLL should involve inhibition of catalytic activity; for the common D427N PKCβ mutant characterised here, this would require development of novel mutant-engaging drugs given the weak activity of current inhibitors. Developments in this direction will be greatly aided by our structural determination of the mutant-inhibitor complex.

The frequent D427N mutation in PKCβ lies within the substrate-binding cleft in the kinase domain [21]. Indeed, many of the *PRKCB* mutations described in ATLL patients lie in or are juxtaposed to this binding pocket [31]. While this might imply strong selection for altered substrate recognition, the autoinhibitory behaviour of the kinase also relies upon this binding cleft, and hence teasing apart altered targets from a degree of constitutive activation is not straightforward, and indeed the combination may well prove key to pathogenesis. Extrapolation from structures of the related PKCι bound to a peptide substrate positions D427 at the P-3 position, a site typically occupied by an arginine residue in primary sequence preferred PKC substrates [32]. The D427N mutant might be expected to lose this preference; however, its ‘PIM kinase-like’ specificity suggests retention of recognition of a P-3 (and P-5) basic residue [33]. Whether the trajectory of substrate interactions is modified in this mutant remains to be determined; however, it is evident from the structural solution of the inhibitor complex that there is very limited variation in the determined kinase domain conformation that would obviously favour altered binding.

The splenomegaly observed in the 18-month-old D427N^het^ mouse cohort was not a consequence of T-cell accumulation but rather due to EMH, as evident from the histological analysis. The finding that the onset of this phenotype was somewhat faster for the homozygous knock-in mice suggests that this PKCβ mutant is indeed a gain-of-function driver for this stress-induced pathology. The lack of any EMH phenotype in the PKCß KO model [13] is consistent with this gain-of-function conclusion. The implication is that the engagement of PKCß in ATLL may be tightly wired by the actions of the predisposing HTLV-1 oncogenes, Tax and/or HBZ.

Consistent with the non-involvement of T-cells in the EMH phenotype and despite the PKCβ D427N mutation being frequent in ATLL, there is a limited impact of the mutant knock-in on the T-cell repertoire, the only significant distinction being in the apparently transient down-regulation of the TCRβ^high^ population in the 12-week-old cohort of heterozygous mutant mice. This may reflect reduced retention of the TCR at the plasma membrane (i.e. through increased internalisation and/or decreased recycling) or a reduction in positive selection. In respect of reduced retention at the plasma membrane, it is of interest that some of the characterised mutations observed in ATLL (GPR183, CCR4 and CCR7) lie on the T-cell trafficking pathway [34]. The non-significant trend towards this TCRβ^high^ population in older mice suggests this is not a key phenotype in respect of the late-onset EMH.

Whilst no definitive statement on PKCβ D427N driver action in ATLL can be made without insight into the underlying molecular mechanism(s), the *in vivo*, cellular and biochemical evidence here indicates that this frequent PKCβ mutation is a gain-of-function mutation albeit with an altered substrate repertoire. In view of the poorer outcomes associated with *PRKCB* mutant disease [12,25], drugs directed at this (mutant) kinase may serve to improve overall survival.

## Materials and methods

### Reagents

Unless otherwise stated, reagents and chemicals were purchased from Sigma-Aldrich.

### Cell lines

HEK239, Jurkat and SF21 cells were obtained from the Cell Services Science Technology Platform at the Francis Crick Institute. HEK293 cells were cultured in DMEM (Thermo Fisher Scientific), and Jurkat cells were cultured in RPMI-1640 (Thermo), both supplemented with 10% FBS (Thermo) and 100 U/ml penicillin-streptomycin (Thermo), at 37°C and 5% CO_2_. SF21 cells were cultured in Sf-900 III SFM (Thermo) supplemented with 1 μg/ml amphotericin B at 27°C (120 rpm).

### Peptides

PSS peptide (CH3CO-ESTVRFARKGSLRQKNVH-CONH2), BTK S180 peptide (CH3CO-RNGSLKPGSSHRKTK-CONH2) and Fluorescein-PSS peptide (FAM-ESTVRFARKGSLRQKNVH-CONH2) were synthesised by the Chemical Biology Science Technology Platform at the Francis Crick Institute and purified by HPLC to >95% purity.

### Plasmids

pEGFP-C1 and pcDNA3 plasmids encoding WT PKCβI and PKCβII were from our laboratory archive. pTriEx-6 empty vector was provided by the Structural Biology Science Technology Platform at the Francis Crick Institute. All other constructs were generated by site-directed mutagenesis (Agilent QuikChange) or In-Fusion Cloning (Takara Bio), according to the manufacturer’s protocols.

### CRISPR/Cas9 reagents

Alt-R S.p. Cas9 nuclease V3 (IDT, #1081058), Alt-R CRISPR-Cas9 sgRNA (IDT, 5′-TGTGATGGAGTATGTGAACG-3′) and Ultramer DNA Oligo (IDT, 5′- CTCTCTTTCCTCAGGACCGCCTGTACTTTGTGATGGAGTATGTGAACGGAGGTAACCTCATGTACCACATCCAACAAGTTGGCCGTTTCAAGGAGCCCCATGCTGTGTAAGACAG-3′) were used to edit *Prkcb* in mice.

### Phosphatidylserine micelles

Brain phosphatidylserine (Avanti Polar Lipids) was dried under nitrogen gas and then resuspended to a concentration of 10 mg/ml in 20 mM Tris-HCl, pH 7.5, containing 1% Triton X-100. The solution was sonicated in a water bath for 10 min.

### HEK293 transfection and protein extraction

HEK293 cells were transiently transfected with purified plasmid DNA using Lipofectamine LTX and PLUS reagent (Thermo), according to the manufacturer’s protocol. After 24–36 hours, cells were lysed in 1% Triton X-100 lysis buffer (130 mM NaCl, 20 mM Tris-HCl pH 8, 1% Triton X-100, 1 mM DTT, 1 mM NaF), supplemented with Complete EDTA-free Protease Inhibitor Cocktail and PhosSTOP (Roche). Lysates were incubated on a rotating wheel at 4°C for 10 min and then centrifuged (13,000× ***g***, 10 min, 4°C). The supernatant was taken for immunoprecipitation or analysed by western blotting.

### Immunoprecipitation kinase assays

Clarified lysates were incubated with GFP-Trap Magnetic Agarose (Chromotek) on a rotating wheel at 4°C for 90 min. The beads were washed 3× in wash buffer (130 mM NaCl, 20 mM Tris-HCl pH 8, 1% Triton X-100, 1 mM DTT, Complete EDTA-free Protease Inhibitor Cocktail) and then pre-incubated in assay buffer (20 mM Tris-HCl pH 8, 2 mM DTT, 10 mM MgCl_2_, 50 μM MBP (Sigma, #M1891), Complete EDTA-free Protease Inhibitor Cocktail and PhosSTOP) with shaking for 15 min at 20°C. Where indicated, assay buffer was supplemented with activators (400 μg/ml phosphatidylserine micelles, 1 μM PMA (Sigma, #P1585) and 300 μM CaCl_2_). The assay was started by adding 300 μM ATPγS (Abcam, #ab138911) and incubated with shaking for 4 min at 20°C. The assay was quenched by adding 20 mM EDTA and then incubated with 5 mM *p*-Nitrobenzyl mesylate (Abcam, #ab138910) for 75 min with shaking at 20°C, before analysis by western blotting.

### Western blotting

NuPAGE 4X LDS Sample Buffer (Thermo) was supplemented with 100 mM DTT and added to samples to 1X concentration. Samples were resolved on NuPAGE 4–12% Bis-Tris gels (Thermo) and transferred to Immobilon-FL PVDF (Millipore). Intercept TBS Blocking Buffer (LI-COR Biosciences) was used to block the membranes and for antibody incubation steps; TBS 0.1% Tween-20 was used for wash steps. The following primary antibodies and dilutions were used: P-PKC T-loop (Cell Signalling Technology (CST), #9379, 1:1000), P-PKC turn motif (CST, #9375, 1:1000), P-PKC hydrophobic motif (CST, #9371, 1:1000), Myc (CST, #2276, 1:1000), HA (CST, #3724, 1:1000), GFP (in-house, 1:2000), thiophosphate ester (Abcam, #ab92570, 1:2000), P(Ser)-PKC Substrate (CST, #2261, 1:1000), PKCβI/βII (BD Biosciences, #610127, 1:500), PKCβII (Santa-Cruz, #sc-13149, 1:1000), GAPDH (Millipore, #MAB374, 1:5000). Fluorescent secondary antibodies (LI-COR Biosciences, #926–68070 and #926–32211) were used at 1:10,000 dilution. The membranes were viewed using an Odyssey CLx machine (LI-COR Biosciences) and quantified in Image Studio Lite v5.2.5 (LI-COR Biosciences).

### RT-qPCR

RNA was extracted from Jurkat cells using the RNeasy Mini Kit (QIAGEN) according to the manufacturer’s protocol. cDNA synthesis, reverse transcription and qPCR were performed as described in [35], with the exception that prior to qPCR the cDNA was cleaned up using a NucleoSpin Gel and PCR Clean-up kit (Thermo). The following qPCR primers were used: PKCβI, 5′- AGCCAAAAGCTAGAGACAAGAGA-3′ (forward) and 5′-GCACCGTGAATCCTGGAAGA-3′ (reverse); PKCβII, 5′-TCTGCAAGGGCTGATGACC-3′ (forward) and 5′-CCTGATGACTTCCTGGTCGG-3′ (reverse); total PKCβ, 5′-CACTCCAGACTACATCGCCC-3′ (forward) and 5′-GGTGTTTGGTCATCAGCCCT-3′ (reverse); actin, 5′-TGGATGAGCAAGCAGGAGT-3′ (forward) and 5′-GCATTTGCGGTGGACCAT-3′ (reverse). The % isoform incidence for PKCβI and PKCβII was calculated from the fold change in expression relative to total PKCβ (2^−ΔCT^ × 100).

### Analysis of PKCβ isoform incidence in ATLL patient samples

PBMCs were isolated from whole blood of two ATLL patients (Supplementary Table S1), following the protocol described by Rowan et al. (2016). Unless otherwise specified, cells were stored in liquid nitrogen with FBS/10% DMSO until use. Total RNA was extracted from PBMCs using the miRNeasy Mini Kit (Qiagen).

RNA-seq libraries were prepared from total RNA using the TruSeq Stranded mRNA HT Sample Prep Kit (Illumina). Paired-end sequencing (150 bp) was performed on the Illumina HiSeq 4000 platform. Sequencing and initial quality control, including alignment to the GRCh37.EBVB95-8wt.ERCC reference genome, were performed by the Oxford Genomics Centre (Wellcome Trust Centre for Human Genetics).

### PKCβ expression and purification

SF21 cells, pTriEx-6 transfer vectors for WT/D427N GST-(3C)-PKCβII (± a TEV site at position 321), *flash*BAC Ultra (Oxford Expression Technologies) and FuGENE reagent (Promega) were used to produce recombinant baculoviruses according to the *flash*BAC manufacturer’s protocol. For expression, SF21 cells at 1 × 10^6^ cells/ml were infected with baculovirus and harvested after 72 h (the volume of virus was chosen to give viability >75% and mean diameter >19 μm at the point of harvest). The cells were resuspended in lysis buffer (50 mM Tris-HCl pH 7.5, 150 mM NaCl, 1 mM EDTA, 1% Triton X-100, Complete EDTA-free Protease Inhibitor Cocktail and 1 mM DTT) and incubated with rotation at 4°C for 1 hour. The soluble fraction was separated by centrifugation (20,000× ***g***, 20 min, 4°C) and incubated with Glutathione-Sepharose 4B beads (GE Healthcare) for 2 hours with rotation at 4°C. The beads were washed under vacuum with wash buffer (50 mM Tris-HCl pH 7.5, 150 mM NaCl, 1 mM EDTA and 1 mM DTT), resuspended in wash buffer and incubated with either GST-3C (to cleave full-length PKCβII; in-house) or His-TEV (to cleave the catalytic domain; in-house) overnight with rotation at 4°C. His-TEV was subsequently removed by the addition of Ni-NTA resin (Thermo). The cleaved protein was further purified on a Superdex 200 Increase 10/300 GL column (Cytiva) equilibrated in gel filtration buffer (50 mM Tris-HCl pH 7.5, 150 mM NaCl, 1 mM EDTA and 0.5 mM TCEP-HCl pH 7, 5% glycerol). Fractions containing pure protein were pooled, concentrated to 0.5–5 mg/ml and stored at −80°C.

### Mass spectrometry

Purified proteins were electrophoresed on NuPAGE 4-12% Bis-Tris gels (Thermo), and the bands were excised, destained, alkylated and digested with trypsin (Promega). Digests were loaded onto Evotips (Evosep), and peptides eluted using the ‘30SPD’ gradient on an Evosep One HPLC fitted with a 15 cm C18 column (Evosep, #EV1074) into a Lumos Tribrid Orbitrap mass spectrometer (Thermo) via a nanospray emitter operated at 2200 V. The Orbitrap was operated in ‘Data Dependent Acquisition’ mode with precursor ion spectra acquired at 120 k resolution in the Orbitrap and MS/MS spectra in the ion trap at 32% HCD collision energy in ‘TopS’ mode. Dynamic exclusion was set to ±10 ppm over 15 s, Automatic Gain Control to ‘standard’ and max. injection time to ‘Dynamic’. The vendor’s ‘universal method’ was adopted to schedule the ion trap accumulation times. Raw files were processed using Maxquant [36] and Perseus [37] with a recent download of the Uniprot *E. coli* reference proteome database together with a common contaminants database. A decoy database of reversed sequences was used to filter false positives, with both peptide and protein false detection rates set to 1%. Skyline [38] was used to quantify peptides using precursor ion extracted ion chromatograms after manual alignment of retention time windows.

### *In vitro* kinase assays

Kinase assays were carried out in white, 384-well OptiPlates (PerkinElmer) using an ADP-Glo kit (Promega) on a scale of 5 ml for the kinase reaction. Kinase was diluted in assay buffer (40 mM Tris-HCl pH 7.5, 20 mM MgCl_2_, 0.1 mg/ml BSA (NEB, #B9000S), 1 mM DTT) and added to the plate (typically 0.5–1 ng/well). The assay was started by adding the substrates at a final concentration of 50–300 μM [ATP and either histone type III-S (Sigma, #H5505), MBP (Sigma, #M1891), or a peptide]. Where indicated, activators were added to the assay buffer [final concentration 400 μg/ml phosphatidylserine micelles, 1 μM PMA (Sigma, #P1585) and 300 μM CaCl_2_]. Where indicated, the kinase was mixed with an inhibitor, ruboxistaurin (Selleckchem, #S7663), BIM-1 (Sigma, #B6292), or UCN-01 (Sigma, #U6508), and in this case 0.02% Triton X-100 was added to the assay buffer to aid solubility. The kinase was assayed for 20 minutes at room temperature before proceeding with the steps described in the ADP-Glo manufacturer’s protocol. Luminescence was recorded using an EnSight MultiMode Plate Reader (PerkinElmer), and the slope of a standard curve was used to convert luminescence values to specific activity in nmol ADP/min/mg.

### Fluorescence polarisation

Fluorescence polarisation (FP) experiments were performed using an FP-8500 spectrofluorometer (Jasco). The excitation wavelength was 498 nm, the emission wavelength was 525 nm and the temperature was 20°C. Titrations were performed by adding small volumes of WT or D427N PKCβII catalytic domains to a cuvette containing 280 nM fluorescein-PSS peptide in FP buffer (50 mM Tris-HCl pH 7.5, 150 mM NaCl, 5 mM MgCl_2_, 0.05% Brij-35 (Thermo), 500 μM AMP-PCP, 5% glycerol). After each addition, the sample was equilibrated for 3–5 min (or until anisotropy was stable) before recording the signal. At least four anisotropy measurements were taken at each concentration of PKCβII and averaged. Equilibrium dissociation constants were determined by plotting anisotropy as a function of PKCβII catalytic domain concentration. The data were fitted assuming a 1:1 interaction, using non-linear least-squares regression with in-house software.

### PamChip® peptide array

WT or D427N PKCβII catalytic domains (2, 20 or 200 ng) were assayed on serine/threonine PamChips (Pamgene, #32501) in a PamStation 12 (Pamgene) according to the manufacturer’s Serine/Threonine Kinase assay protocol. Reagents were from the serine/threonine reagent kit (Pamgene, #32201). PamChips were first blocked with 2% BSA. An assay mix containing kinase and a serine/threonine primary antibody was added. The PamChips were then washed with PBS 0.01% Tween, and detection was carried out using an anti-rabbit Alexa-Fluor 488 nm secondary antibody at 20 mg/ml (Thermofisher, #A11008). Data analysis was done in BioNavigator (Pamgene). Images taken at different exposure times were integrated to give a single signal value, and the log2 fold change relative to the no ATP control array was calculated for peptides which passed quality control steps. The BioNavigator Upstream Kinase Analysis tool was used, in which publicly available databases are used to predict kinase activity on the array compared with the control.

### D427N PKCβ mouse model

All mice were housed in the Biological Research Facility at the Francis Crick Institute, and studies were done in accordance with a project licence granted by the Home Office and animal welfare guidelines. Mice were culled by cervical dislocation. No procedures were done on live mice. No anaesthetics were used. Studies were performed under the Procedure Project Licences 77/8066. Mice were maintained on a C57BL/6J background. CRISPR/Cas9 was used to generate the D427N PKCβ mouse model. The guide RNA was designed using CRISPOR [39] to cut at site chr7:122,181,684 in exon 11 of *Prkcb*. The donor DNA template was designed with 53 bp of homology upstream of the guide cut site and 61 bp downstream, as well as a silent mutation to mutate the PAM site. Zygotes were generated by superovulation of C57BL/6 J mice and electroporated with 1.2 μM Cas9/6 mM guide RNA/8 μM donor DNA [40]. Both template and guide were added at the one-cell stage. Electroporation of the embryos was performed using a Nepa21 electroporator (Sonidel). Transnetyx qPCR was performed on mouse ear snips to identify nine F_0_ mice preliminarily positive for the mutation. A total of 500 bp around the donor sequence was amplified by PCR and sequenced by the Advanced Sequencing Facility at the Francis Crick Institute using Illumina MiSeq 250 bp paired-end reads. Three F_0_ mice had sequence-perfect integration of the D427N mutation. One F_0_ mouse was bred to C57BL/6J, which resulted in three F_1_ mice identified as heterozygous for the mutation by Illumina MiSeq sequencing and one copy present by PCR copy number evaluation. One founder was also sequenced up to 1606 bp upstream and 1757 bp downstream of the mutation with no aberrant off-target effects. F_1_ mice were backcrossed to C57BL/6J.

### Spleen harvest, flow cytometry and histopathology

Mice were culled by a schedule one method and the spleen was taken for processing. A quarter of the spleen was fixed in 10% neutral buffered formalin for 24 h at room temperature and then processed on a Tissue-Tek VIP 5 tissue processor, embedded in paraffin wax (Leica, #3,808,605E) and sectioned on a Leica RM microtome at 3 μm. The slides were loaded onto a Tissue-Tek Prima autostainer for staining with haematoxylin (Leica, #3,801,590E) and eosin (Leica, #3,801,560E) according to standard protocols. The remainder of the spleen was crushed through a 70 μM filter in RPMI medium. The cells were pelleted (300× ***g***, 5 min, 4°C) and resuspended in PBS/1% FBS. They were stained with antibodies at 1:100 dilution in PBS/1% FBS for 30 min at 4°C in the dark, washed with PBS/1% FBS, resuspended in 0.1 mg/ml DAPI (BD Biosciences; #564907) in PBS/1% FBS, filtered and analysed on an LSRFortessa (BD Biosciences). The antibodies used were BD Biosciences B220-APC (#561880), CD4-PE-Cy7 (#552775) and CD8-APC (#553035). Data analysis was done in FlowJo v10.7.2 (BD Life Sciences).

### Crystallography and structure determination

D427N PKCβII catalytic domain was concentrated to 4.8 mg/ml in 50 mM Tris-HCl pH 7.5, 150 mM NaCl, 0.5 mM TCEP-HCl pH 7, 5 mM MgCl_2_, 0.1 mM EDTA and 5% glycerol. Ruboxistaurin (Selleckchem, #S7663) was added at 242 μM final concentration. Vapour diffusion crystallisation screens were set up in MRC 2-drop trays at 22°C using a Mosquito robot (TTP LabTech). Rod-shaped crystals of D427N PKCβII catalytic domain grew in 0.1 M trisodium citrate pH 5.17 + 12.3% w/v PEG 6K. The crystals were mounted in a Hampton loop, dipped into mother liquor supplemented with 20% ethylene glycol and frozen in liquid nitrogen. Data were collected at Diamond Light Source on the I24 beamline. Due to the small size of the crystals (150 × 7 × 7 µm), data were collected on a CdTe Eiger2 9M detector using X-rays at a wavelength of 0.67 Å to allow us to exploit the photoelectron escape effect [41]. The beam size was matched approximately to the minimum width of the crystals (7 × 7 μm) and a line scan was performed to minimise radiation damage whilst maximising the diffracted intensity. 360° of data were collected with an image width of 0.1° (3600 images) and 0.2 s exposure per image travelling along the length of the crystal (150 μm). The data were indexed, integrated and scaled using DIALS [42]. The data extended to 3.4 Å resolution, the crystals had P3_1_21 symmetry, and the unit cell dimensions were a=102.33 Å, b=102.33, c=82.83 Å, a=90°, b=90°, g=120°. The Matthews coefficient [43] suggested that there was one copy in the asymmetric unit (60% solvent content). The CCP4 suite was used for data processing [44]. The structure was solved by molecular replacement using Phaser [45] in the MrBUMP molecular replacement pipeline [46]. The best solution was obtained using a structure of rat PKCβII [47]. The molecular replacement solution had a translation function Z-score of 30.9 and after refinement with jelly-body restraints in REFMAC [48], the model had an R_free_ of 35%, indicating that a correct solution had been found. After successive rounds of rebuilding in Coot [49] and refinement in REFMAC, the data-to-model correlation was improved to a final R_free_ of 29.0%. In the penultimate step of rebuilding, the inhibitor ruboxistaurin (PDB LY4) was imported from a structure of ruboxistaurin-bound PIM1 [50] and fitted to the difference density in the nucleotide-binding pocket. Data collection and refinement statistics are summarised in Supplementary Table S2.

## Supplementary material

Online supplementary material 1

## Data Availability

The authors affirm that all the data supporting the findings of this study are available within the article and supplementary materials. The structure of the ruboxistaurin inhibitor bound to PKCβII kinase domain has been deposited at the PDB Databank with the accession code 9S9T.

## Abbreviations

ATLL: adult T-cell leukaemia/lymphoma
BTK: Bruton’s tyrosine kinase
EMH: extramedullary haematopoiesis
FBS: Fetal Bovine Serum
FSC-A: Forward Scatter Area
GFP: Green fluorescent protein
HA: hemagglutinin
HCD: Higher-energy collisional dissociation
HEK293: Human embryonic kidney 293 cells
HTLV-1: Human T-cell leukaemia virus type 1
IC50: half-maximal inhibitory concentration
MBP: myelin basic protein
PBMCs: peripheral blood mononuclear cells
PKCβ: protein kinase C beta
PMA: phorbol-12-myristate 13-acetate
SD: Standard deviation
SF21: Spodoptera frugiperda cell line 21
SSC-A: Side Scatter Area
cPKC: conventional PKC
hets: heterozygotes
qPCR: Quantitative Polymerase Chain Reaction
