# Supplementary material for: Penetrant PKCβ mutation in ATLL displays a mixed gain-of-function
Source: Biochem J. 2025 Nov 4;482(21):1659–75. doi: 10.1042/BCJ20253384 (PMC12687424; doi:10.1042/BCJ20253384)
Supplement: Online supplementary material 1 [file bcj-482-21-BCJ20253384-s001.docx]

**SUPPLEMENTARY FIGURE 1**

**
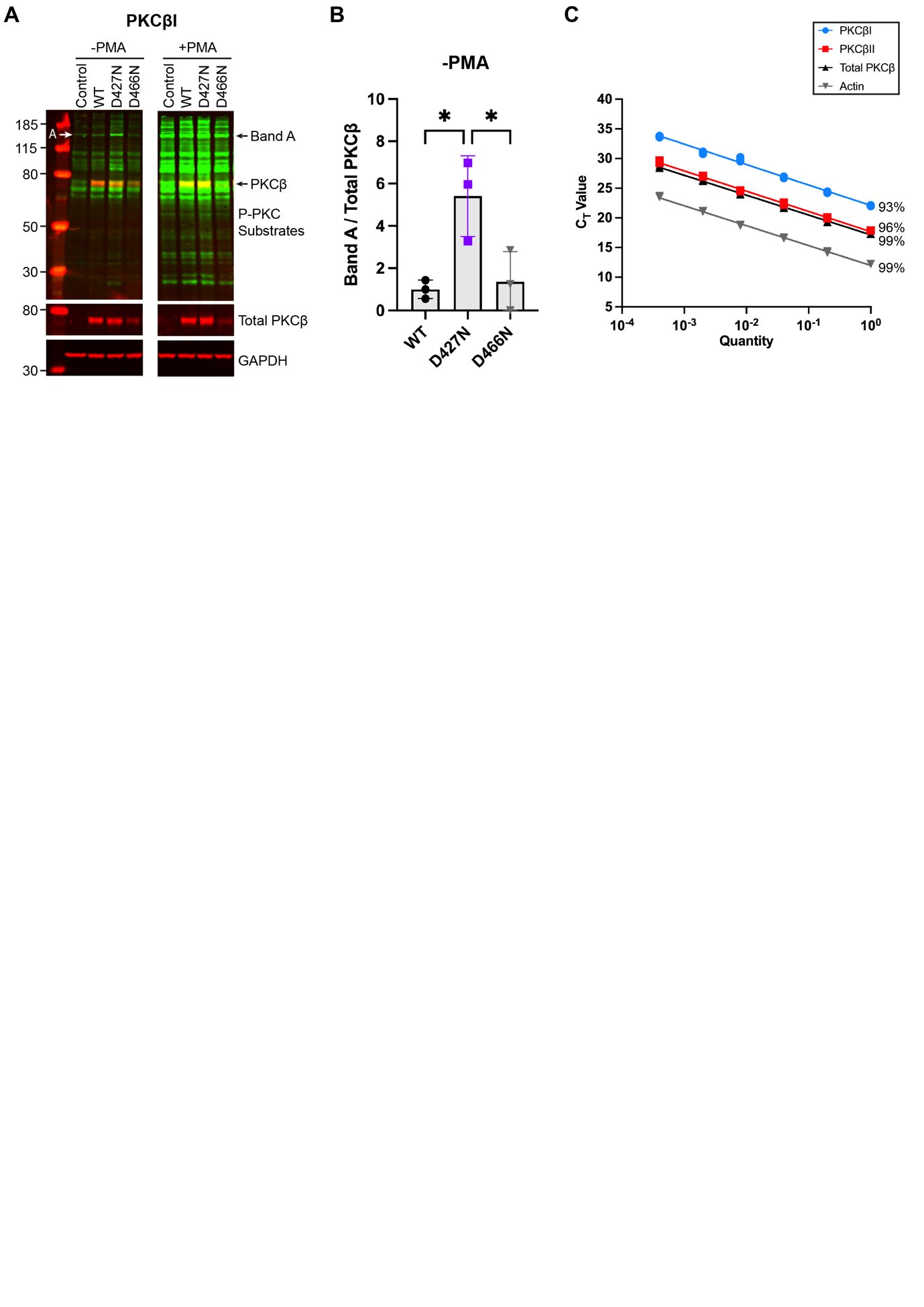
**

**Figure S1. Phosphorylated PKC substrates in PKCβI-transfected HEK293 cells, and qPCR controls. A** – Western blots of lysates from HEK293 cells transfected with the indicated PKCβI constructs and treated ± PMA for 30 min prior to lysis. The blot was probed with antibodies against total PKCβ, GAPDH and phosphorylated serine residues at a PKC consensus site. The control was untransfected cells and the blots are representative of three biological replicates. **B** – Quantification of band A from untreated cells in (A). Band A signal in each lane was first normalised to GAPDH. The normalised control lane value was then subtracted from the analysis. The ratio of band A to total PKCβ was then calculated; values are shown relative to WT PKCβI and error bars indicate mean ± SD (n=3, *=p<0.05 versus WT by unpaired t-test). **C** – % efficiency for each qPCR primer. A serial dilution of Jurkat cDNA was analysed by qPCR and the slope used to calculate efficiency for each primer pair, $E= \left( {10}^{-1/slope}-1 \right) \times100$.

**SUPPLEMENTARY FIGURE 2**


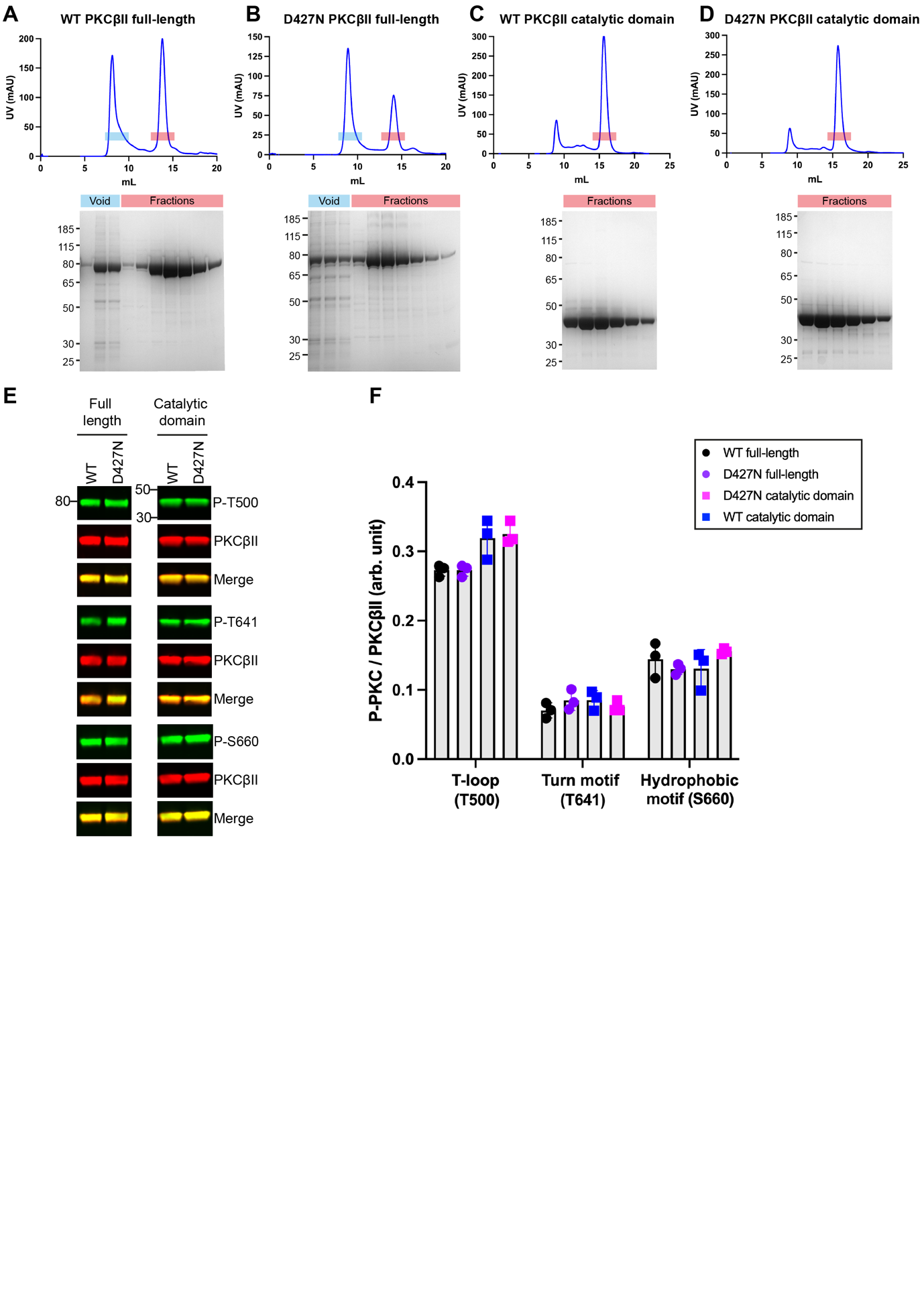


**Figure S2. Purification and priming of WT and D427N PKCβII (full-length and catalytic domains). A – D** – Representative chromatograms monitoring absorbance at 280 nm during gel filtration of each protein, alongside Coomassie-stained SDS-PAGE gels of the indicated fractions. **E** – Western blots of purified proteins, probed with antibodies for each phosphorylated PKC priming site and total PKCβII. Blots are representative of three independent preparations. **F** – Quantification of the ratio of phosphorylated/total PKCβII for each priming site from the blots in (E). Error bars indicate the mean ± SD from three independent preparations.

**SUPPLEMENTARY FIGURE 3**

**
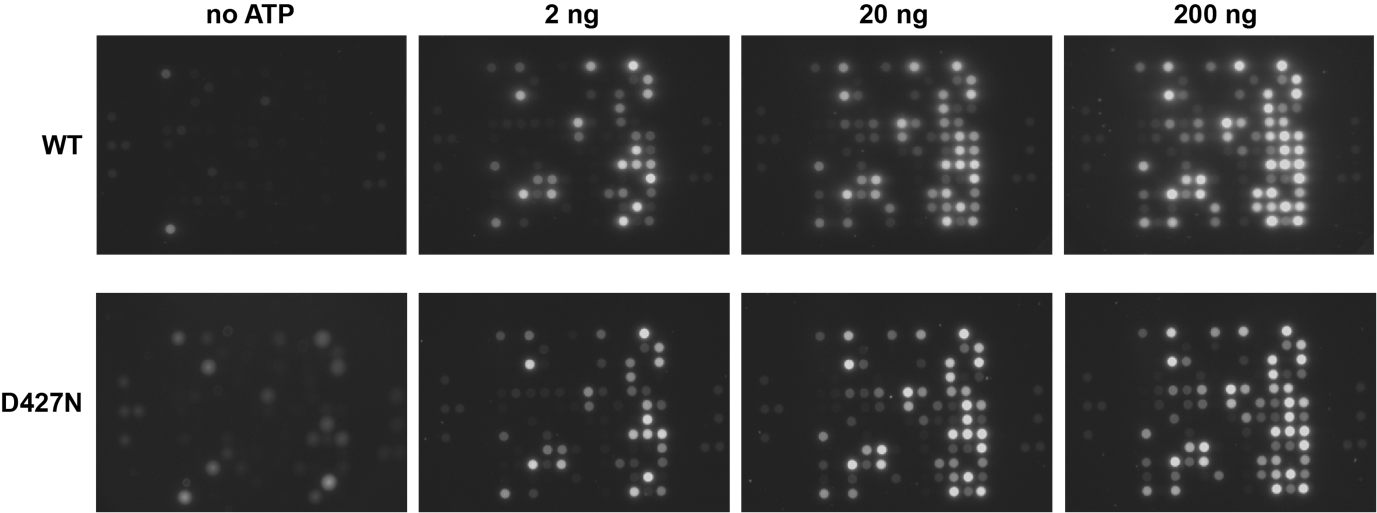
**

**Figure S3. PamChip serine/threonine microarrays phosphorylated by WT and D472N PKCβII catalytic domains.** In a PamStation, different quantities of WT or D427N PKCβII catalytic domains were incubated with ATP on a serine/threonine PamChip. 20 ng of kinase was also incubated without ATP as a control. PamChips were then incubated with a mixture of anti-phospho serine/threonine antibodies and phosphorylation was detected with a fluorescently labelled secondary antibody.

**SUPPLEMENTARY FIGURE 4**

**
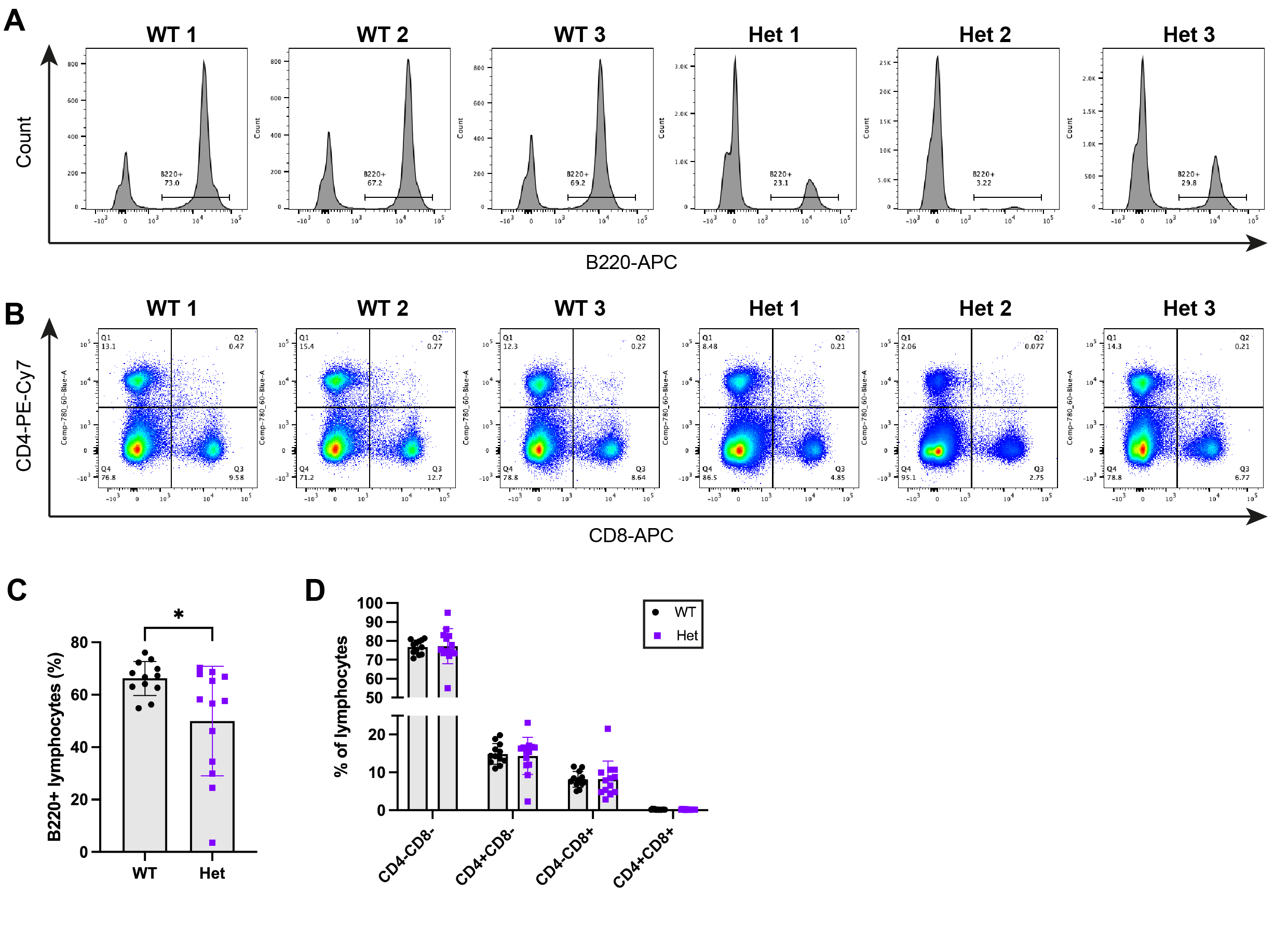
**

**Figure S4. Proportion of B and T-cells in the spleen of a D427N PKCβ mouse model.** Spleens were harvested from WT and D427N PKCβ hets at 18 months old and a single cell suspension was analysed by FACS. Mouse numbers correspond to Fig 4. **A** – Histograms of B220 expression on the live lymphocyte population. **B** – Log dot plots showing CD4 vs CD8 expression on the live lymphocyte population. **C** – % of B220+ lymphocytes in the spleen. Error bars indicate the mean ± SD from 12 mice (*=p<0.05 by unpaired t-test with Welch’s correction). **D** – % of CD4 and CD8 single, double positive and double negative lymphocytes. Error bars indicate the mean ± SD from 12 mice.

**SUPPLEMENTARY TABLE 1**

| **Protein** | **Evosep/Lumos**  **+P/total** | | **U3000/Q-Exactive +P/total** | **Combined**  **+P/total** |
| --- | --- | --- | --- | --- |
|  | **2+ ion** | **3+ ion** | **3+ ion** |  |
| **T-loop** | | | | |
| **WT full length** | 0.71 | 0.64 | N/A | 0.69 |
| **WT catalytic domain** | 0.35 | 0.43 | N/A | 0.40 |
| **D427N full length** | 0.84 | 0.86 | N/A | 0.85 |
| **D427N catalytic domain** | 0.74 | 0.77 | N/A | 0.76 |
| **Turn motif** | | | | |
| **WT full length** | 0.72 | 0.80 | 0.72 | 0.77 |
| **WT catalytic domain** | 0.83 | 0.89 | 0.83 | 0.89 |
| **D427N full length** | 0.99 | 0.99 | 0.99 | 0.99 |
| **D427N catalytic domain** | 0.99 | 0.99 | 0.99 | 0.99 |
| **Hydrophobic motif** | | | | |
| **WT full length** | N/A | N/A | 0.90 | 0.90 |
| **WT catalytic domain** | N/A | N/A | 0.93 | 0.93 |
| **D427N full length** | N/A | N/A | 0.93 | 0.93 |
| **D427N catalytic domain** | N/A | N/A | 0.95 | 0.95 |

**Table S1. Purified WT and D427N PKCβII priming stoichiometry determined by MS/MS.** Data were collected with two different LC-MS/MS systems (Evosep-Lumos or U3000-Q-Exactive). Intensities for phosphorylated vs unphosphorylated peptides were determined in Skyline. The phosphorylation stoichiometry (+P/total) was calculated for each system and either the 2+ or 3+ peptide ions. Where data are missing, it was not possible to calculate stoichiometry. The final column shows a combined stoichiometry from all the available data.

**SUPPLEMENTARY TABLE 2**

**Table 1. Data collection and refinement statistics.**

|  | **PDB 9S9T** |
| --- | --- |
| **Wavelength** | 0.67Å |
| **Resolution range** | 60.51 - 3.426Å (3.92 - 3.43ÅÍ) |
| **Space group** | P 31 2 1 |
| **Unit cell** | 102.327Å 102.327Å 82.827Å 90 90 120 |
| **Total reflections** | 140696 (47355) |
| **Unique reflections** | 7006 (2286) |
| **Multiplicity** | 20.1 (20.7) |
| **Completeness (%)** | 99.64 (99.08) |
| **Mean I/sigma(I)** | 3.42 (0.54) |
| **Wilson B-factor** | 125.57 |
| **R-merge** | 0.4482 (1.968) |
| **R-meas** | 0.46 (2.018) |
| **R-pim** | 0.1025 (0.4417) |
| **CC1/2** | 0.992 (0.631) |
| **CC*** | 0.998 (0.88) |
| **Reflections used in refinement** | 6986 (2270) |
| **Reflections used for R-free** | 362 (122) |
| **R-work** | 0.2142 |
| **R-free** | 0.2897 |
| **Number of non-hydrogen atoms** | 2564 |
| **macromolecules** | 2529 |
| **ligands** | 35 |
| **solvent** | 0 |
| **Protein residues** | 315 |
| **RMS(bonds)** | 0.018 |
| **RMS(angles)** | 2.14 |
| **Ramachandran favored (%)** | 93.71 |
| **Ramachandran allowed (%)** | 6.29 |
| **Ramachandran outliers (%)** | 0.00 |
| **Rotamer outliers (%)** | 2.26 |
| **Clashscore** | 0.60 |
| **Average B-factor** | 145.75 |
| **macromolecules** | 145.96 |
| **ligands** | 130.04 |

Statistics for the highest-resolution shell are shown in parentheses.

**Table S2. X-ray crystallography data collection and refinement statistics.**
